# Atlas der Klinischen Mikroskopie des Blutes

**Published:** 1908-12

**Authors:** 


### Reviews of Books.

**Atlas der Klinischen Mikroskopie des Blutes.** Zweite auflage, bearbeitet von Privatdocent Dr. E. MEYER and Professor Dr. H. RIEDER. Leipzig: F. C. W. Vogel. 1907.

This atlas consists of a series of plates, beautifully executed in colours, and one or two in monochrome, of the microscopic appearances of the blood in disease. A short, but clear and adequate, descriptive letterpress, with explanatory diagrams relating to the coloured plates, is also given. It forms a most valuable help to the student beginning the study of blood-diseases, and a useful atlas for reference to those more advanced in this work.

